# Analysis of prognostic factors and construction of prognostic models for triple-positive breast cancer

**DOI:** 10.3389/fonc.2023.1071076

**Published:** 2023-02-01

**Authors:** Anqi Geng, Jingjing Xiao, Bingyao Dong, Shifang Yuan

**Affiliations:** Department of Thyroid, Breast and Vascular Surgery, Xijing Hospital, Fourth Military Medical University, Xi’an, China

**Keywords:** triple positive breast cancer, prognostic model, nomogram, overall survival, SEER

## Abstract

**Objective:**

By identifying the clinicopathological characteristics and prognostic influences of patients with triple-positive breast cancer (TPBC) at Xijing Hospital in China compared with those in the United States, this study aims to construct a nomogram model to forecast the overall survival rate (OS) of TPBC patients.

**Method:**

The Surveillance, Epidemiology, and End Results (SEER) database was used to screen 5769 patients as the training cohort, and 191 patients from Xijing Hospital were used as the validation cohort. Cox risk-proportional model was applied to select variables and the nomogram model was constructed based on the training cohort. The performance of the model was evaluated by calculating the C-index and generating calibration plots in the training and validation cohorts.

**Results:**

Cox multifactorial analysis showed that age, chemotherapy, radiotherapy, M-stage, T-stage, N-stage, and the mode of surgery were all independent risk factors for the prognosis of TPBC patients (all P<0.05). With this premise, the nomogram model was constructed and evaluated. The C-index value of the nomogram model was 0.830 in the training group and 0.914 in the validation group. Moreover, both the calibration and ROC curves for the proposed model exhibited reliable performance, and the clinical decision curve analysis showed that the proposed model can bring clinical benefits.

**Conclusions:**

The constructed nomogram can accurately predict individual survival probabilities and may serve as a clinical decision support tool for clinicians to optimize treatment in individuals.

## Introduction

Breast cancer comprises a highly diverse set of systemic illnesses on a molecular level. According to the 2011 St Gallen International Expert Consensus on the Primary Therapy of Early Breast Cancer, breast cancer can be classified into four subtypes based on immunohistochemical evaluation of hormone receptors (HRs), including the estrogen receptor (ER), progesterone receptor (PR), and human epidermal growth factor receptor 2 (HER2) (1). Among these subtypes is the triple-positive breast cancer (TPBC) subtype, which is immunohistochemically expressed as ER+/PR+/HER2+ and any Ki-67 status and accounts for approximately 10% of all breast cancer cases (2).

Individuals with TPBC were found to have higher tumor grades, larger tumors, and poorer prognoses than those with other subtypes, and their tumors exhibited aggressive behavior (3). To date, only a few studies have explicitly focused on the clinical features and prognosis of TPBC. According to Anderson et al., the age of onset of TPBC was concentrated between 45 and 75 years, and the prognosis was poorer than that of HR(+) and HER-2(-) subtypes but better than that of HER2-enriched ones (4). Treatment typically consists of adjuvant chemotherapy combined with trastuzumab, followed by endocrine therapy in TPBCs that express both hormonal receptors and HER2 (5). Although patients benefit from multiple treatment options, interactions among various treatment regimens may reduce the therapeutic impact, most likely due to crosstalk between the HER-2 and ER gene signaling pathways at multiple points (6). Additionally, You et al. observed that the overall survival rate of patients with TPBC was higher than the survival rate of those with HER2-enriched ones and similar to those with the luminal A subtype (7).

For many years, the prognosis of patients with breast cancer has been assessed using the TMN prognostic staging method. The eighth edition of the American Joint Committee on Cancer’s prognostic staging method integrates the state of estrogen and progesterone receptors, HER2 expression, and histological grading based on TNM staging (8). Its prognostic value and availability in patients with breast cancer have been validated since the development of this revolutionary breast cancer staging system. However, the prognostic staging system appears quite complex for clinical application due to the recurrent grouping (9–12). Furthermore, because genetic testing technology is still not extensively employed, the new system’s clinical usefulness is limited. In addition, breast cancer is highly heterogeneous and the individual prognosis is affected by a wide range of factors (13). According to He et al. (14), the novel prognostic staging approach did not outperform the anatomical staging system in terms of prediction power for triple-negative breast cancer. Adjustment and optimization of the prognostic staging system are still needed. Hence, building adequate predictive models for the various molecular subtypes of breast cancer can benefit clinical practice.

The Surveillance, Epidemiology, and End Results (SEER) database is one of the most representative large tumor registry databases in North America, collecting a large amount of relevant data on evidence-based medicine and covering approximately 1/3 of the US population. The nomogram is a commonly used method for survival prediction that combines intuition, accuracy, dependability, and practicality (15). It has been successfully used to predict the prognosis of various malignancies, including breast cancer (16). In this study, data from the Fourth Military Medical University Affiliated Xijing Hospital and the SEER database were synthesized to construct a nomogram model for predicting the overall survival (OS) of TPBC patients, aiming to provide a basis for clinical treatment.

## Patients and methods

### Patient selection

**Figure 1:**
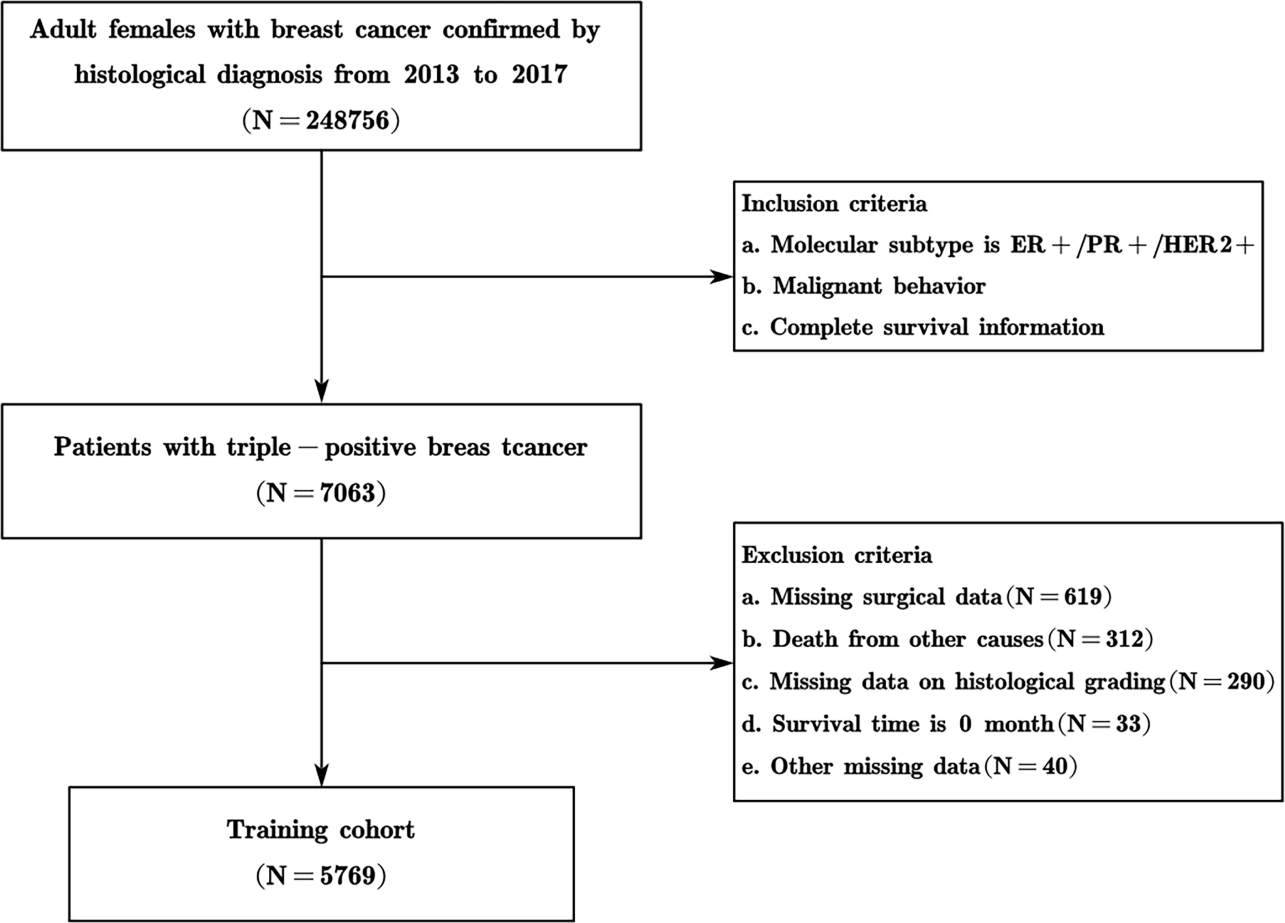
Flowchart of the cohort selection process.

SEER is a large-scale cancer registration database that covers approximately 34.6% of the U.S. population (17). The data for this study were selected from 17 registries of the SEER program (with an additional treatment field), which is supported by the National Cancer Institute. The data of TPBC patients from January 2013 to December 2017 in the SEER database were extracted and screened by SEER*Stat version 8.4.0.1 software. The inclusion criteria were as follows: (1) patients with pathologically confirmed breast cancer, based on malignant behavior of the International Classification of Diseases (ICD)-O-3; (2) female; (3) molecular subtype is ER+/PR+/HER2+; (4) older than 18 years; (5) survival data with complete and available dates and more than 0 days of survival; and (6) clear clinicopathological information for all the variables of interest including age at diagnosis, breast subtype, tumor size, laterality, lymph node metastasis status, distant metastatic status, type of surgery, pathological type, histological grading, chemotherapy, and radiotherapy information. According to the inclusion and exclusion criteria, cases meeting the criteria were gradually screened out, and 5769 patients with TPBC were ultimately included (**Figure 1**). Moreover, 191 patients with triple-positive breast cancer at Xijing Hospital in China hospitalized for surgery from January 2013 to December 2017 were collected, and the clinicopathological characteristics and prognosis of the patients were determined. Follow-up was performed by in-hospital review, telephone consultations, and instructional activities.

### Study variables

The following variables at diagnosis were selected as the potential prognostic factors: age(less than 60 years old or older than 60 years old), laterality (right or left side), pathological type (infiltrative ductal carcinoma or other types), histological grading (well-differentiated, moderately differentiated, poorly differentiated, undifferentiated or anaplastic), tumor size, lymph node metastasis status, distant metastatic status, type of surgery, chemotherapy (yes or none) and radiotherapy (yes or none). The values of tumor size, lymph node metastasis status, distant metastasis status, and surgery type were transformed into grouped categorical variables according to routine practice.

Overall survival (OS) was used as the primary endpoint for this study. OS was defined as the time between the date of diagnosis and the date of death caused by BC. For the validation cohort, the deadline for follow-up was September 14, 2021.

### Statistical analysis

The data were analyzed using R software (4.1.1). All variables were transformed into categorical variables. The baseline characteristics of the modeled and validated sets were compared using the Pearson χ^2^ test, where the Mann−Whitney U test was performed for the rank data. The Kaplan−Meier curve was used to describe the OS, and the differences between the curves were analyzed by the log-rank test. Univariate and multivariate Cox regression models were performed to estimate the hazard ratios (HRs) and 95% confidence intervals (CIs) to analyze the independent prognostic factors associated with OS in TPBC patients. Based on the independent prognostic factors of TPBC, the Rms and Survival packages in R software (4.1.1) were used to construct the nomogram. To confirm the predictive accuracy of the nomogram, both internal (200 bootstraps resamples based on the training cohort) and external (based on the validation cohort) validations were performed. The differentiation of the model was evaluated by the concordance index (C-index) and the receiver operating characteristic (ROC) curve, and the calibration of the model was checked by drawing calibration curves to ensure that the model was accurate and reliable. Finally, decision curve analysis (DCA) was performed for the model to check the clinical benefit and application value of the model. Two-sided P<0.05 was deemed statistically significant.

## Results

### Baseline characteristics of TPBC patients

The Pearson χ^2^ test and Mann−Whitney U test were used to compare the baseline characteristics of the training and validation sets. The results showed that the patients in the validation set were younger at onset and had a lower proportion of poorly differentiated histology compared to the training set. In addition, there were significant differences in pathological staging, tumor size, lymph node metastasis, distant metastasis, and choice of treatment modality (all p<0.05) (**Table 1**).

**Table 1:** Baseline characteristics of TPBC patients.

Characteristics	Training cohort (n=5769)	Validation cohort (n=191)	χ^2^ value	P value
	Number of patients (%)	Number of patients (%)		
Age				
<60	3376 (58.5)	168 (86.6)	61.37	<0.001
≥60	2393 (41.5)	26 (13.4)		
Laterality				
Left	2998 (52)	103 (53.1)	0.095	0.758
Right	2771 (48)	91 (46.9)		
Pathological type				
Infiltrative ductal carcinoma	4909 (85.1)	191 (98.5)	27.068	<0.001
Other types	860 (14.9)	3 (1.5)		
Histological grading				
Grade I	386 (6.7)	7 (3.6)		<0.001^a^
Grade II	2575 (44.6)	146 (75.3)		
Grade III+IV	2808 (48.7)	41 (21.1)		
T				
T≤2	3170 (54.9)	140 (72.2)		<0.001^a^
2<T≤5	2050 (35.5)	46 (23.7)		
5<T	549 (9.6)	8 (4.1)		
N				
0	3822 (66.3)	115 (59.4)		<0.003^a^
1	1495 (25.9)	42 (21.6)		
2	300 (5.2)	22 (13.3)		
3	152 (2.6)	15 (7.7)		
M				
0	5655 (98)	175 (90.2)	52.603	<0.001
1	114 (2)	19 (9.8)		
Type of surgery				
Total mastectomy	1692 (29.3)	35 (18)	311.515	<0.001
Breast-conserving surgery	3287 (57)	43 (22.2)		
Modified radical surgery	790 (13.7)	116 (59.8)		
Chemotherapy				
None	1232 (21.4)	26 (13.4)	7.132	0.008
Yes	4537 (78.6)	168 (86.6)		
Radiotherapy				
None	2403 (41.7)	119 (61.3)	29.804	<0.001
Yes	3366 (58.3)	75 (38.7)		

^a^Mann-Whitney U test.

### Effect of variables on the prognosis of TPBC

For each variable in the training set, a COX univariate survival analysis was performed. The results showed that nine variables, including age, tumor grade, radiotherapy, chemotherapy, pathological staging, T-stage, N-stage, M-stage, and mode of surgery, were factors influencing the prognosis of TPBC (all p<0.05). Multivariate analysis using Cox proportional risk regression (variable screening method: forward: LR, variable inclusion criterion = 0.05, exclusion criterion = 0.1) was performed with the above risk factors as independent variables, and the results indicated that age, radiotherapy, chemotherapy, T-stage, N-stage, M-stage, and mode of surgery were independent risk factors for the prognosis of TPBC (all p<0.05) (**Table 2**).

**Table 2:** Univariate and multivariate analysis of TPBC patients.

Characteristics	Univariate analysis		Multivariate analysis	
	HR(95%CI)	P value	HR(95%CI)	P value
Age				
<60	1.00	<0.001	1.00	<0.001
≥60	1.93(1.50-2.57)		1.86(1.37-2.53)	
Histological grading				
Grade I	1.00	0.002		
Grade II	1.15(0.57-2.31)			
Grade III+IV	1.89(0.96-3.72)			
T				
T≤2	1.00	<0.001	1.00	<0.001
2<T≤5	3.35(2.33-4.81)		2.63(1.77-3.89)	
5<T	8.37(5.65-12.40)		4.27(2.68-6.80)	
N				
0	1.00	<0.001	1.00	<0.001
1	3.11(2.19-4.41)		2.96(1.99-4.42)	
2	8.62(5.70-13.03)		5.59(3.44-9.09)	
3	12.31(7.83-19.35)		6.68(3.83-11.64)	
M				
0	1.00	<0.001	1.00	<0.001
1	6.66(4.23-10.48)		2.47(1.52-4.00)	
Type of surgery				
Total mastectomy	1.00	<0.001		<0.001
Breast-conserving surgery	0.63(0.43-0.91)		0.87(0.58-1.31)	
Modified radical surgery	3.50(2.47-4.98)		1.60(1.08-2.38)	
Chemotherapy				
None	1.00	<0.001	1.00	<0.001
Yes	0.43(0.32-0.57)		0.28(0.20-0.39)	
Radiotherapy				
None	1.00	0.001		0.005
Yes	0.63(0.47-0.84)		0.58(0.42-0.80)	

**Figure 2:**
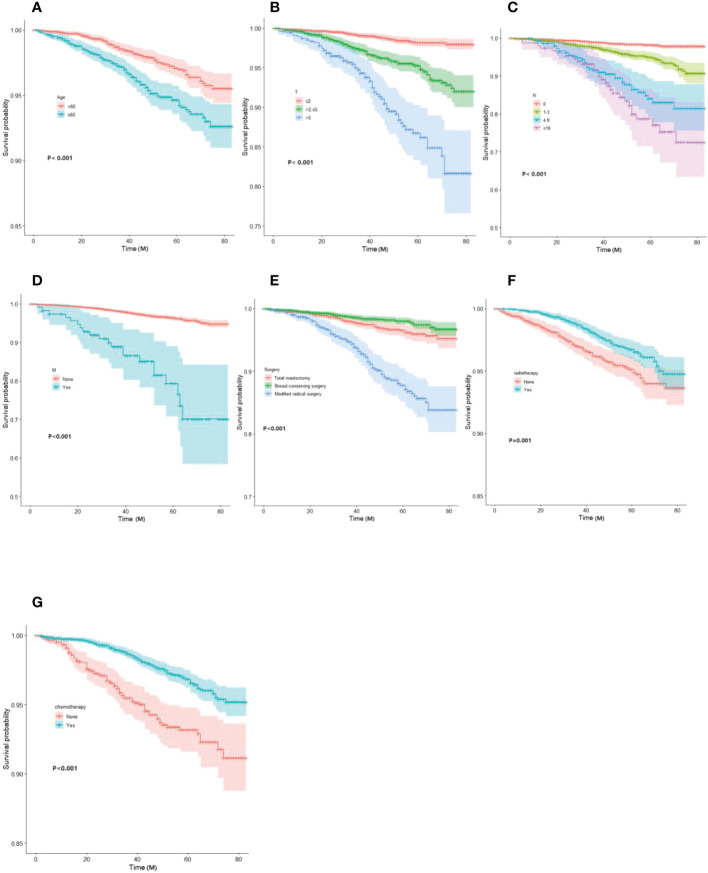
Kaplan-Meier curves of OS for each predictor. **(A)** Age; **(B)**T-stage; **(C)** N-stage; **(D)** M-stage; **(E)** mode of surgery; **(F)** radiotherapy; **(G)** chemotherapy. OS, overall survival.

Based on Kaplan-Meier and Log-rank tests, the survival curves for the key variables were plotted using the Cox risk model (**Figure 2**).

### Construction of a nomogram for the prognosis of TPBC patients

**Figure 3:**
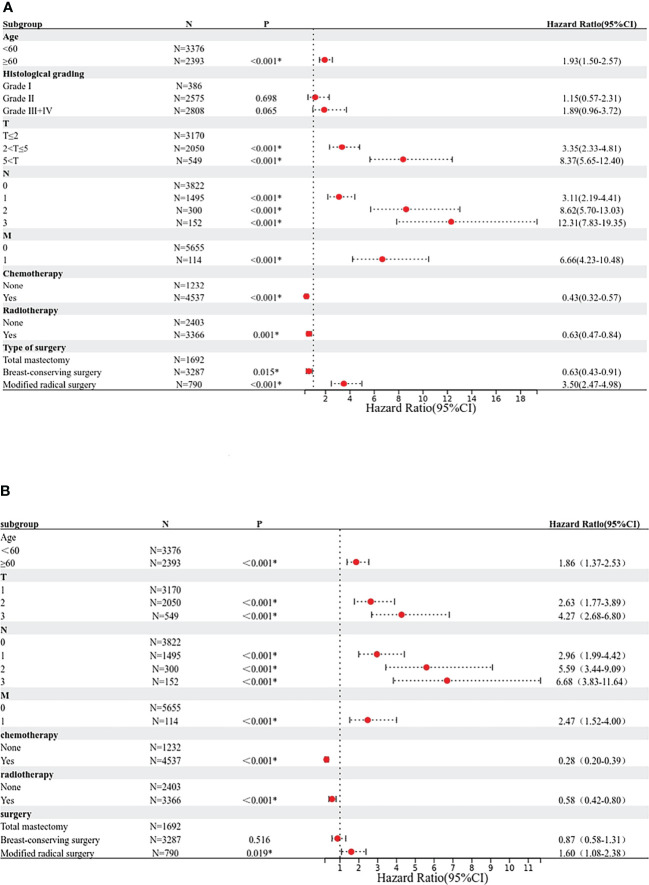
Proportional hazard model forest map of overall survival in TPBC patients in SEER. **(A)** Forest map (univariate analysis). **(B)** Forest map (multivariate analysis). * means p < 0.05.

**Figure 4:**
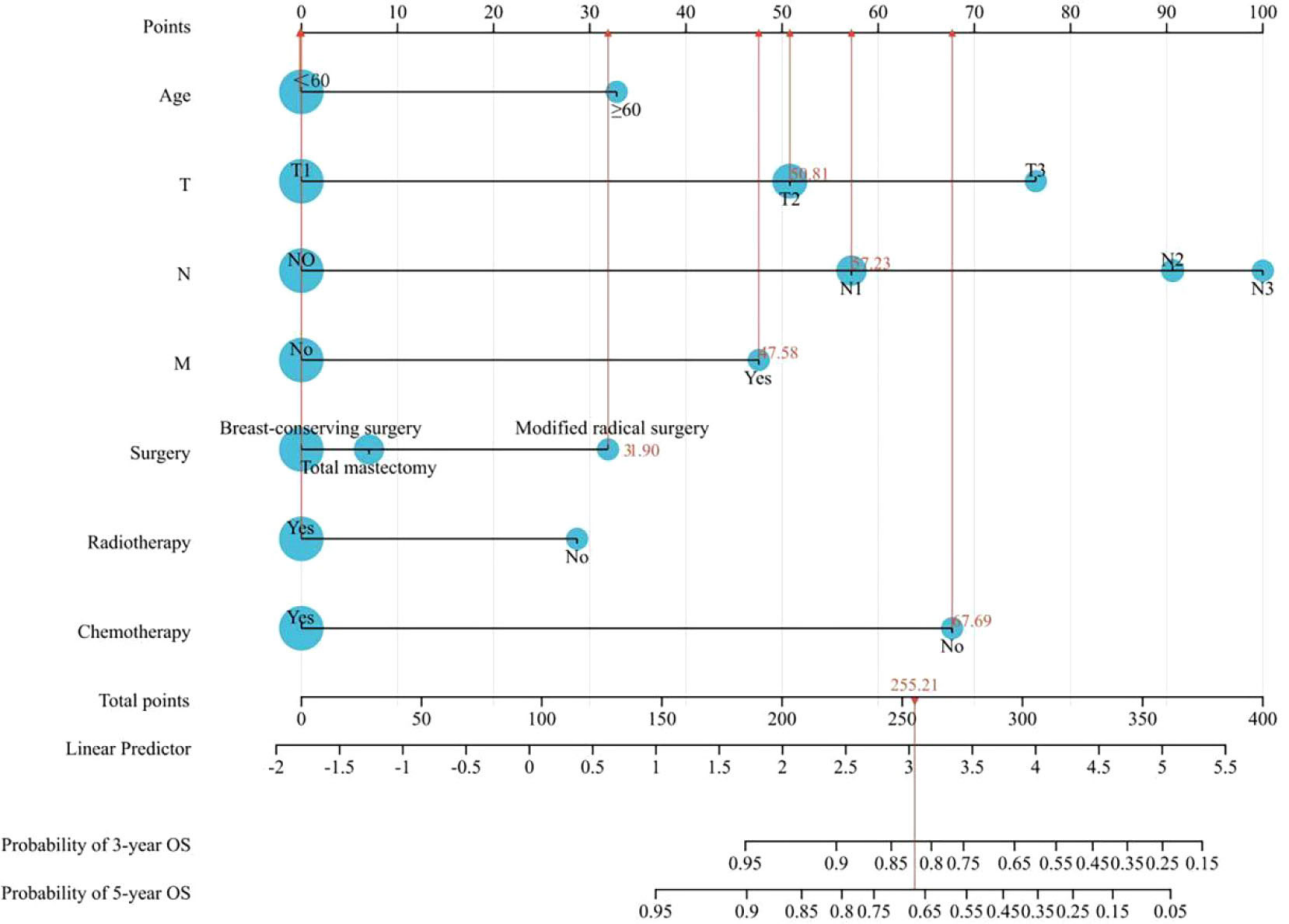
Nomogram prediction model for prognosis of TPBC patients.

Based on the results of the Cox univariate and multivariate regression analysis of the training group (**Figures 3A, B**), the seven variables screened were used to construct a nomogram of the OS prognosis of 5769 TPBC patients (**Figure 4**). By summing the scores obtained for each variable to obtain an overall score, the nomogram prediction model predicts the 3-year and 5-year OS for TPBC patients.

### Validation of the nomograms

**Figure 5:**
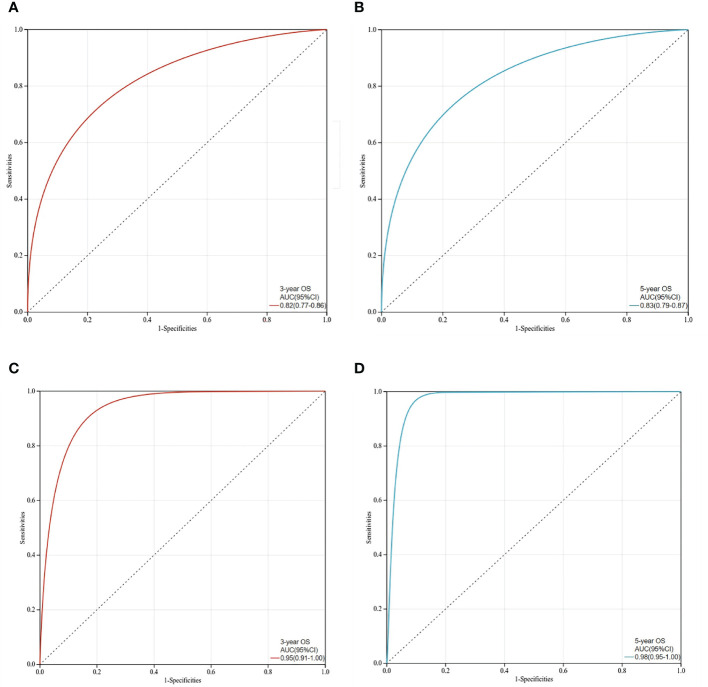
ROC curves for prediction of 3-year and 5-year overall survival in the training set and validation set. **(A)**The 3-year overall survival of the training set; **(B)**The 5-year overall survival of the training set; **(C)** The 3-year overall survival of the validation set; **(D)** The 5-year overall survival in the validation set.

**Figure 6:**
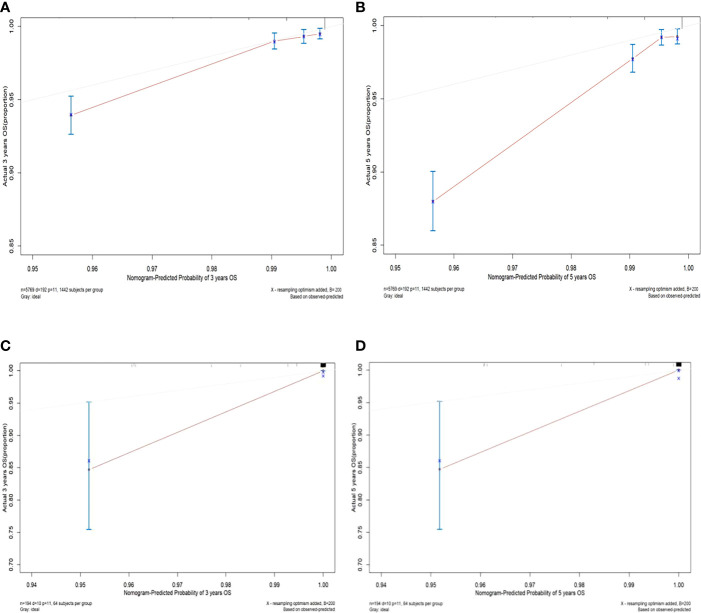
Calibration curves of 3-year and 5-year overall survival in the training set and validation set. **(A)** The 3-year overall survival of the training set; **(B)** The 5-year overall survival of the training set; **(C)** The 3-year overall survival of the validation set; **(D)** The 5-year overall survival in the validation set.

The C-index values of the nomograms in the training cohort were 0.830 (95% CI, 0.795-0.864) for OS. In the validation cohort, the C-index value for OS was 0.914 (95% CI, 0.816-0.999). In addition, the ROC curves and calibration curves of the 3-year and 5-year OS were plotted in the training and validation sets. The results showed that the area under the curve (AUG) was greater than 0.8 in both the training and validation sets, while the calibration curves presented excellent consistency between the actual and nomogram-predicted survival probabilities, indicating that the model predicted with decent accuracy (**Figures 5**, **6**).

### DCA analysis

**Figure 7:**
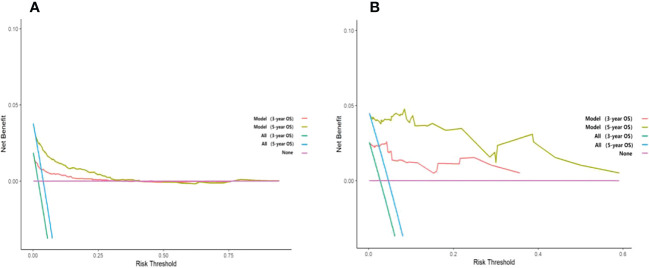
Decision curve analysis curves of 3-year and 5-year overall survival in the training set and validation set. **(A)** The OS of the training set; **(B)** The OS of the validation set.

Unlike traditional statistical methods, which only evaluate the accuracy of a model, decision curve analysis (DCA) can tell us whether using a model to aid clinical decision-making would improve outcomes for our patients (18). In this study, DCA was plotted against 3-year and 5-year survival for the training and validation sets, respectively. The results show that the net clinical benefit of the model at 3 and 5 years is elevated within a suitable threshold in both the training and validation sets, especially in the validation set, indicating that the model has excellent clinical efficacy (**Figure 7**).

## Discussion

TPBC is a subtype of breast cancer that falls within the luminaI B molecular type (19), accounting for approximately 10% of hormone receptor-positive breast cancers (20). Currently, there are few studies on the prognosis of triple-positive breast cancer. Kast et al. (21) stated that TPBC is aggressive cancer, with ductal carcinoma being the most common. Pathologically, most cases were classified as grade III, with an elevated prevalence of lymph node metastases and giant tumors. Additionally, Guan et al. showed that patients with TPBC tended to be younger and exhibit pathological characteristics such as vascular or nerve infiltration and an elevated rate of lymph node metastases, proliferation index, and tumor load (22). In this study, TPBC patients had a younger age of onset, a higher percentage of histological grade III, and significant lymph node metastases, consistent with previous studies. In summary, triple-positive breast cancer is a relatively aggressive molecular subtype.

Since TPBC is a relatively rare and clinically neglected condition, assessment of the prognosis of patients with TPBC is essential for the integrated management of TPBC. Numerous studies have investigated breast cancer prognosis, and molecular type, tumor size, lymph node status, and histological grading are often used as prognostic indications in clinical practice. Additionally, while both the 21-gene and 70-gene recurrence scores are approved for clinical use, the use of multiple testing to predict recurrence remains contentious because of the limited clinical benefit and extreme cost (16). There is currently a shortage of simple and effective prognostic and predictive assessment methods that may be used in clinical practice. No acceptable model for prognostic assessment has been created in prior investigations, especially for TPBC. The nomogram is a graphical representation of the multivariate prognostic model, which can be used to individually predict the survival situation at a specific time point (23). As a contemporary forecasting model, nomograms have higher accuracy and wider applicability and are easy to popularize compared with traditional forecasting methods (24). As reported in the literature, Zhou et al. constructed and validated well-calibrated nomograms for predicting disease-free survival and OS in patients with TNBC (25). In addition, as one of the largest cancer registries in the United States, the SEER database contains a wealth of evidence-based medical data, including basic information, clinical characteristics, treatments, and patient follow-up. Therefore, this study developed a prognostic prediction nomogram model for TPBC patients based on data from the SEER database, which is reduplicative.

In this study, we developed a nomogram-based Cox regression model to predict the 3-year and 5-year OS of TPBC patients. The ROC and calibration curves showed that the nomogram could accurately predict the OS of TPBC patients. At the same time, decision curve analysis showed that the clinical efficacy of the model was excellent. Multivariate analysis showed that age, tumor grade, radiotherapy, chemotherapy, stage T, stage N, stage M, and surgical modality were independent risk factors for TPBC prognosis. These independent risk factors were essentially consistent with clinical observations.

It has been shown that HR-positive, HER2-positive breast cancer patients older than 75 years have significantly increased mortality compared to other populations (26). Our findings also suggested a poor prognosis for TPBC patients over the age of 60. Currently, TNM prognostic staging is commonly used to assess the prognosis of breast cancer patients. In this study, patients had a worse prognosis as the TNM stage increased, which is consistent with previous studies (27). Breast-conserving therapy (BCT) had similar long-term survival outcomes to mastectomy in patients with early breast cancer, and recent studies had reported similar rates of recurrence compared with mastectomy (28). However, the latest research showed that BCT was associated with superior overall survival compared with mastectomy for early-stage breast cancer (29), consistent with this study. In addition, by comparing data, we observed a relatively elevated BCT rate in the United States. The reason lies in the uniformity of diagnosis and treatment levels and treatment standards among American doctors and in the fact that doctors can follow treatment standards very well.

Theoretically, endocrine therapy, chemotherapy, and targeted therapy have a significant impact on the prognosis of patients with TPBC. In 2017, NCCN guidelines recommended chemotherapy in combination with anti-HER-2 therapy and endocrine therapy as a treatment regimen for TPBC (30). Targeted therapy is vital in the adjuvant treatment of HER2-positive early-stage breast cancer. The clinical trial HERA study revealed that 79.4% of patients survived for >10 years and were at a lower risk of death after 1 year of trastuzumab adjuvant therapy (31). However, in clinical practice, we found that patients with TPBC had less benefit from trastuzumab, which may have been due to drug resistance. Studies have demonstrated that the presence or absence of HRs is a crucial component in determining breast cancer diagnosis, therapy, and prognosis (32). A secondary analysis (33) of the HERA study published in 2016 confirmed the lower benefit of trastuzumab in patients with TPBC with high ER expression. This was demonstrated by the interaction between ERs and the intracellular signaling pathway regulated by HER-2 (34).

Although other researchers have done similar work (27), Compared with previous studies, the innovations of this study are as follows. Firstly, by including a Chinese cohort, the variability of clinical characteristics of TPBC patients by race was explored. Secondly, to externally validate the model, this study used a Chinese cohort, and the results were more compelling. Finally, to improve the model’s construction and validation, this study included survival analysis and clinical decision curves.

Through internal and external validation, our constructed nomogram showed excellent accuracy and clinical benefit. However, there were still some limitations to this study. First, due to the limitations of the data in the SEER database, the predictive model cannot include some crucial clinical factors, such as chemotherapy protocol, targeted therapy regimen, endocrine therapy regimen, and Ki-67 expression, and additional studies may be needed to optimize the model. At the same time, 1294 of the 7063 identified TPBC patients were excluded due to insufficient data, which may have contributed to selection bias. Furthermore, the constructed nomogram had only been externally validated with a single sample in China, so caution should be exercised in extending our results to patients from different geographic regions or with other ethnic backgrounds.

## Conclusion

In summary, this study developed a nomogram model to predict the overall survival of TPBC patients based on data from the SEER database in the United States and Xijing Hospital in China. Both the calibration curves and ROC curves for the model exhibited reliable performance, and the clinical decision curve analyses showed that the model can bring clinical benefit. Therefore, the constructed nomogram can accurately predict individual survival probabilities and may serve as a clinical decision support tool for clinicians to optimize treatment in individuals.

## Data availability statement

The datasets presented in this study can be found in online repositories. The names of the repository/repositories and accession number(s) can be found in the article/supplementary material.

## Ethics statement

Ethical review and approval was not required for the study on human participants in accordance with the local legislation and institutional requirements. Written informed consent for participation was not required for this study in accordance with the national legislation and the institutional requirements.

## Author contributions

Conception and design, SY. Collection and assembly of data, JX. Acquisition of study materials or patients, BD. Data analysis and interpretation, AG. Manuscript writing, AG. Final approval of manuscript, all authors. All authors contributed to the article and approved the submitted version.
